# Prehabilitation Before Gastrointestinal Cancer Surgery: Protocol for an Implementation Study

**DOI:** 10.2196/41101

**Published:** 2023-03-27

**Authors:** Kristy-Lee Raso, Michael Suen, Jane Turner, Sonia Khatri, Yanlan Lin, Carolyn Wildbore, Guillermo Becerril-Martinez, Philip Le Page, Sim Yee Tan, Sam Egger, Janette Vardy

**Affiliations:** 1 Department of Nutrition and Dietetics Concord Repatriation General Hospital Concord Australia; 2 Sydney Medical School University of Sydney Sydney Australia; 3 Department of Colorectal Surgery Concord Repatriation General Hospital Concord Australia; 4 Concord Cancer Centre Concord Repatriation General Hospital Concord Australia; 5 Department of Upper Gastrointestinal Surgery Concord Repatriation General Hospital Concord Australia; 6 The Daffodil Centre, The University of Sydney, A Joint Venture with Cancer Council New South Wales Sydney Australia

**Keywords:** prehabilitation, gastrointestinal, cancer, surgery, exercise, nutrition, telehealth, colorectal, psychological

## Abstract

**Background:**

Surgery remains the standard curative treatment for early-stage colorectal and upper gastrointestinal cancer. Reduced preoperative functional capacity, nutritional status, and psychological well-being are associated with poor postoperative outcomes. Prehabilitation aims to improve preoperative functional reserves through physical, nutritional, and psychological interventions. Yet, how it transitions from a trial setting to being integrated into a real-world health setting is unknown.

**Objective:**

The primary aim is to evaluate the implementation of a multimodal (supervised exercise, nutrition, and nursing support) prehabilitation program into standard care for patients with gastrointestinal cancer (colorectal and upper gastrointestinal cancer) scheduled for curative intent surgery. The secondary aim is to determine the impact of a multimodal prehabilitation program on functional capacity, nutritional and psychological status, and surgical outcomes.

**Methods:**

This is an implementation study that will investigate a multimodal prehabilitation intervention, in a nonblinded, nonrandomized, single-group, pre-post design. Patients diagnosed with colorectal and upper gastrointestinal cancer scheduled for potentially curative intent surgery at Concord Repatriation General Hospital, with ≥14 intervention days prior to surgery and are medically cleared to exercise will be eligible. The study will be evaluated using the Reach, Effectiveness, Adoption, Implementation, and Maintenance Evaluation Framework.

**Results:**

The protocol was approved in December 2019 by the Concord Repatriation General Hospital Human Research Ethics Committee (reference number 2019/PID13679). Recruitment commenced in January 2020. In response to the COVID-19 pandemic, recruitment was paused in March 2020 and reopened in August 2020 with remote or telehealth intervention adaptations. Recruitment ended on December 31, 2021. Over the 16-month recruitment period, a total of 77 participants were recruited.

**Conclusions:**

Prehabilitation represents an opportunity to maximize functional capacity and improve surgical outcomes. The study will provide guidance and contribute to the evidence on the integration of prehabilitation into standard care using adaptive models of health care delivery including telehealth.

**Trial Registration:**

Australian and New Zealand Clinical Trials Registry ACTR 12620000409976; https://anzctr.org.au/Trial/Registration/TrialReview.aspx?id=378974&isReview=true

**International Registered Report Identifier (IRRID):**

RR1-10.2196/41101

## Introduction

Gastrointestinal cancer is a common type of cancer, with around 28,600 Australians diagnosed each year [1], and incidence rates are expected to increase with our aging population. In Australia alone, colorectal cancer (CRC) is the fourth most diagnosed cancer and the second most common cause of death, with 15,540 new cases and 5295 deaths estimated per year [1]. Upper gastrointestinal (UGI) cancers including esophageal, gastric, hepatic, pancreatic, and biliary represent approximately 12,434 new cancer cases [2] and more than 17% of cancer-associated deaths [1]. Despite improvements in surgical and oncological treatments, morbidity rates remain high, particularly among those with UGI cancer [3].

Surgery remains the best chance of cure for patients with colorectal cancer and upper gastrointestinal cancer. However, even with advances in surgery, anesthesia, and perioperative care, including the application of enhanced recovery after surgery protocols to mitigate the surgical stress response [4,5], postoperative complications still occur in 30%-50% of patients and are associated with higher morbidity and mortality rates, increased health care costs, and reduced quality of life [6-8]. Even in the absence of complications, major gastrointestinal surgery can still result in a 20%-40% reduction in physical and functional capacity, nutritional decline, and delayed recovery [9,10] particularly in the elderly or those with comorbidities, with many not returning to their baseline function or proceeding to their intended oncological treatment.

In the past decade, the focus of optimizing patients in the preoperative period to withstand the upcoming surgical stressor has emerged in a concept known as prehabilitation. Prehabilitation aims to improve preoperative functional reserves through physical, nutritional, and psychological interventions to mitigate the predictable detrimental effects of surgery [11,12]. While early prehabilitation studies focused on the feasibility of singular interventions [12], a multimodal (exercise, nutrition intervention, and psychological support) approach is increasingly favored [13-17]. We previously reported a pilot feasibility study of a short-term multimodal (exercise, healthy eating, and nursing support) prehabilitation program in CRC surgery [18]. In total, 22 participants completed the program, and the median length of the intervention was 14 (range 7-29) days. We found that attendance was excellent (78.5%, mean 3.5 sessions), moderate-intensity aerobic exercise increased from 17 to 73 minutes per week, and functional capacity measured using the 6-minute walk test (6MWT) [19,20] had a clinically significant increase of 48 (from 435 to 483) m. Participant satisfaction was excellent, with all participants strongly recommending prehabilitation.

While the evidence surrounding prehabilitation grows, how it transitions from a trial setting to being integrated into a real-world health setting is less clear. Implementation science frameworks aim to help understand the barriers and facilitators to support the uptake of research findings into routine clinical care [21]. The “RE-AIM” (Reach, Effectiveness, Adoption, Implementation, Maintenance) evaluation framework [22,23] is a theoretical implementation framework that can guide and assess the translation from research into practice at both the individual and organizational levels. The primary aim of this study is to evaluate the implementation of a multimodal (supervised exercise, nutrition intervention, and nursing support) prehabilitation program into standard care for patients with gastrointestinal cancer (CRC and UGI cancer) scheduled for curative intent surgery using the RE-AIM framework. The secondary aim is to determine the impact of a multimodal prehabilitation program on functional capacity, nutritional and psychological status, quality of life (QOL), length of stay at the hospital, and postsurgical outcomes including complications and recovery. We hypothesize that prehabilitation (1) will be feasible, with good participant adherence; (2) will decrease the length of stay at the hospital and postsurgery complications; and (3) improve functional and nutritional status, recovery, and QOL. Here, we outline the trial protocol, including adaptations made to accommodate the COVID-19 pandemic.

## Methods

### Study Design and Setting

This protocol describes a translational, implementation study that will investigate a multimodal prehabilitation intervention, in a nonblinded, nonrandomized, single-group, pre-post design, in patients with colorectal cancer and upper gastrointestinal cancer undergoing potentially curative intent surgery at Concord Repatriation General Hospital, a tertiary teaching hospital in Sydney, Australia. The study will be evaluated using the RE-AIM Evaluation framework [22,23] (Table 1). Exploratory comparisons of some secondary end points will be made with historical colorectal cancer and upper gastrointestinal patient data obtained from the hospital’s surgical database. The minimum recruitment period will be 12 months. Recruitment commenced in January 2020.

**Table 1:** Reach, Effectiveness, Adoption, Implementation, and Maintenance evaluation framework adapted to prehabilitation before gastrointestinal cancer surgery study.

Domain	Description	Data collection instrument
Reach	The number (and proportion) of eligible patients having gastrointestinal cancer surgery who participated (numerator) and how representative they are of the target population (denominator) of participants and eligible patients who did not consent Participant characteristics and comparison to nonparticipants	Patient screening logs Prehabilitation before gastrointestinal cancer surgery study Research Electronic Data Capture (Vanderbilt University) database including demographics
Effectiveness	Changes in functionality, nutrition, and well-being from pre- to post intervention	Participant-reported outcomes (psychological status, and quality of life) and functional assessments (6-minute walk test and 2-minute step test), nutritional status, and body composition, at baseline, before surgery, and 30 days after surgery assessments
Adoption	For the single site, the number of surgeons who participated versus invited surgeons, characteristics of participating surgeons versus non-participating surgeons, the number of referrals received, referral sources, and barriers to referrals Clinician and surgeon perceptions	Number of referring surgeons (numerator) versus total number of surgeons at the site treating colorectal cancer or upper gastrointestinal cancer patients (denominator) In-house clinician satisfaction surveys
Implementation	Fidelity of intervention in the real world: consistency of the intervention delivery described in the protocol and adaptations made Intervention completion rates, defined as the proportion of participants that completed the intervention with reasons for withdrawal described Participant adherence to the intervention and completion of program evaluation	Intervention session notes compared with the protocol Prehabilitation before gastrointestinal cancer surgery study database regarding adherence, completion, and withdrawals Exercise attendance and support phone call logs Self-reported exercise and protein diary Satisfaction surveys and exit interviews
Maintenance	Setting level: extent of program delivery as part of standard care 12 months after study completion Standard care program adaptations	Participant enrolment in the standard care prehabilitation program and delivery as recorded in the medical notes Multimodal program intervention

### Ethics Approval

Ethics approval was granted by Concord Repatriation General Hospital Human Research Ethics Committee under reference number 2019/PID13679, and the study was registered on the Australian and New Zealand Clinical Trials Registry (ACTR 12620000409976). The study will be performed in accordance with the National Health and Medical Research Council National Statement on Ethical Conduct in Human Research (Commonwealth of Australia, 2007 and updates) and the World Medical Assembly (Helsinki 2017 and updates).

### Study Population

Patients will be considered eligible if they are 18 years or older, undergoing elective colorectal cancer or upper gastrointestinal cancer resection with curative intent at the Concord Repatriation General Hospital with a minimum of 14 days prior to surgery for the intervention. All participants must be medically fit to exercise as determined by their treating physician, willing to attend supervised exercise sessions at the Sydney Cancer Survivorship Gym Concord Repatriation General Hospital 1-2 times per week, take prescribed high protein oral nutritional supplements, and have Eastern Co-operative Oncology Group performance status of 0-2 [24]. Patients treated with neoadjuvant chemotherapy or radiotherapy are eligible to participate after completion of neoadjuvant chemotherapy or radiotherapy provided there is a 14-day intervention time available before surgery. Patients who do not speak English are eligible and will be provided with a professional health interpreter. Inclusion and exclusion criteria are further outlined in Textbox 1.

**Study inclusion and exclusion criteria.**

**Inclusion criteria**

- Aged at least 18 years
- Give written informed consent
- Confirmed stage I-III colorectal or upper gastrointestinal cancer or limited stage IV (eg, limited liver metastases planned for curative resection) cancer that is potentially curable with surgery
- Elective surgery scheduled ≥14 days after the time of referral
- Medically cleared to exercise, as determined by the treating physician or Adult Pre-exercise Screening System page 1 V2 (2019) [25]
- Willing to attend supervised exercise sessions at the Concord Repatriation General Hospital Sydney Cancer Survivorship Gym 1-2 times per week until surgery
- Willing to take daily high protein study oral nutritional supplement until surgery
- Eastern Co-operative Oncology Group [24] performance status of 0-2
- Willing to complete patient-reported outcome questionnaires and exercise or dietary logs
- Agreeable to follow-up for 30 days after surgery

**Exclusion criteria**

- Unable to exercise or take high protein oral nutritional supplements for physical or medical reasons
- Taking immunonutrition supplements (eg, oral nutritional supplements with immunomodulating ingredients) during the intervention
- Unable to give informed consent or follow instructions due to cognitive difficulties
- Currently receiving chemotherapy or radiotherapy (patients will be eligible providing ≥14 days is still available after completion of neoadjuvant chemotherapy and radiotherapy and before surgery)

### Study Procedure

**Figure 1:**
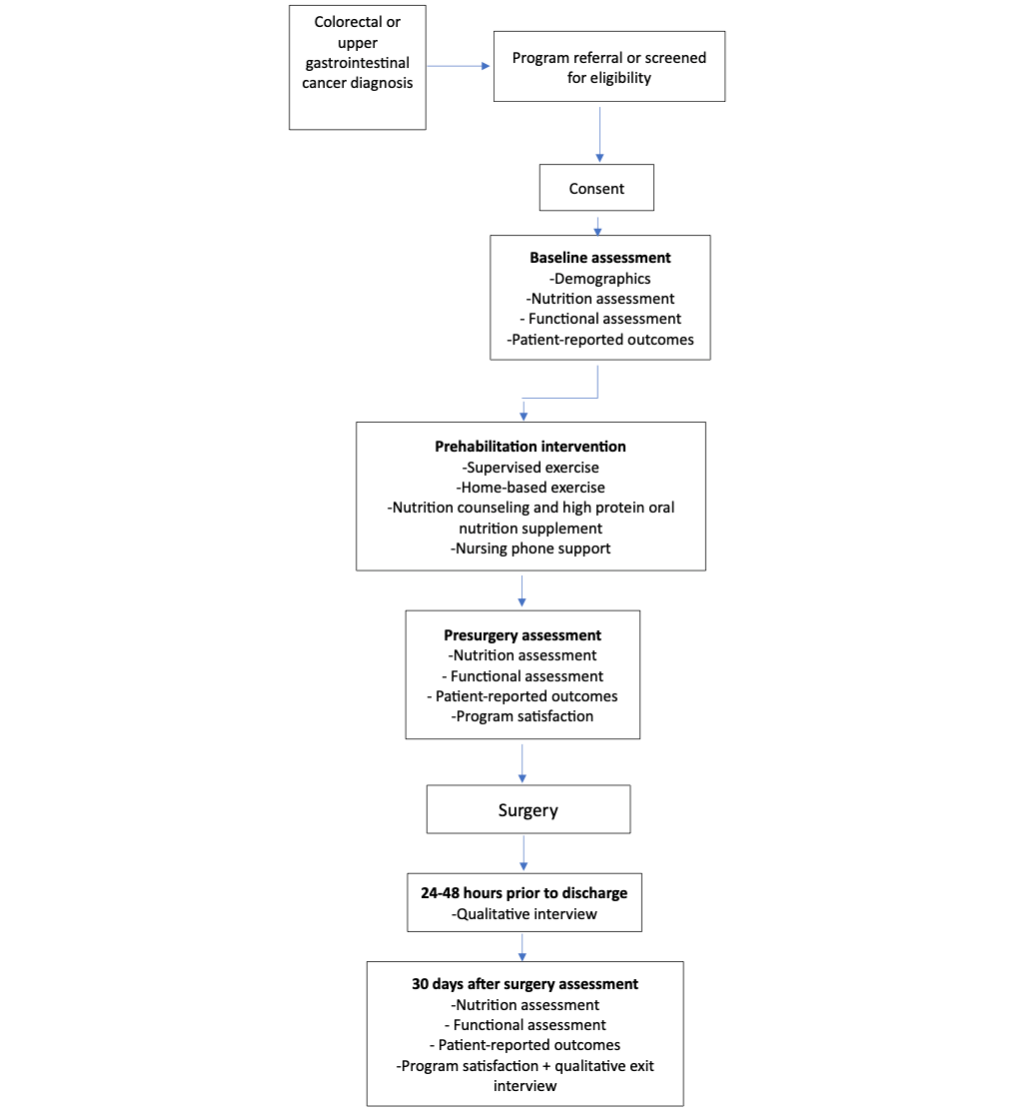
Study flowchart.

Patients will be referred directly by their surgeon or oncologist or screened in a preadmission clinic for eligibility. Patient flow throughout the study is presented in Figure 1. The study coordinator or cancer nurse specialist will screen and contact eligible patients to explain the study. After consent, a baseline assessment will be conducted in eligible participants within 1-2 days of referral from the surgeon or preadmission clinic visit. Day 1 of the intervention period will be the day of the baseline assessment.

### Intervention

**Figure 2:**
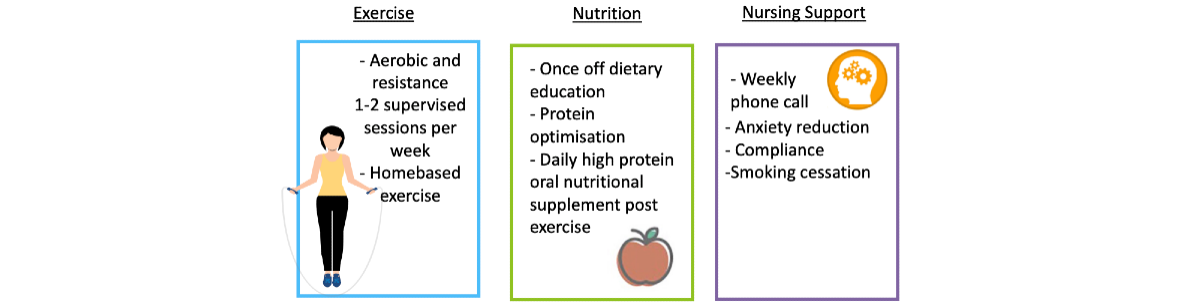
Multimodal prehabilitation intervention.

The duration of the intervention will vary depending on the scheduled surgery date but will run over 2-4 weeks (minimum of 14 days). The intervention is outlined in Figure 2.

### Exercise

An accredited exercise physiologist will assess the participant’s mobility and exercise capacity during the baseline assessment. Participants must commit to a minimum of one 60-minute weekly supervised session but will be encouraged to attend two 60-minute supervised exercise sessions per week.

Exercise sessions will include both aerobic and resistance exercise training. These sessions will be individually prescribed, facilitated, and monitored by an accredited exercise physiologist at the Sydney Cancer Survivorship Gym, Concord Cancer Centre. Exercise sessions will be delivered either individually or in a small group-based format with other cancer survivors. All participants will complete a warm-up routine consisting of dynamic stretching and a range of motion exercises. Participants will complete 20 minutes of aerobic exercise per 60-minute session with a target intensity of 60%-75% of heart rate reserve. Aerobic exercise may include the use of a cycle ergometer, treadmill, rowing ergometer, elliptical trainer, boxing due to equipment availability, and will be selected depending on participants’ preference and capability. The remainder of each session will focus on resistance exercises targeting large muscle groups through multijoint movements and use body weight, hand weights, resistance bands, or cable machine exercises. Participants are assisted in completing 2 sets of 8-12 repetitions for each movement with a target rating of perceived exertion of 13-15, measured using Borg’s Rating of Perceived Exertion 6-20 scale [26]. Heart rate and rating of perceived exertion will be recorded throughout each session for safety monitoring and exercise prescription purposes. The exercise volume and intensity will be adjusted by the exercise physiologist in a stepwise fashion.

Participants will be advised to participate in a nonhospital–based aerobic exercise (eg, walk, jog, cycle, swim, and step) program tailored to their baseline ability on at least 5 days per 7-day period. The target duration is a minimum of 30 minutes (continuous or broken into shorter bouts) per day at a self-rated perceived exertion of moderate intensity (13-15). An exercise diary and instructions will be provided to capture the frequency, intensity, time, and type of exercise. During the supervised exercise sessions, the exercise physiologist will provide support to promote program compliance, progression, and adherence.

### Nutrition

Nutritional assessments will be completed by an Accredited Dietitian at baseline, before surgery, and 30 days after surgery, using The Patient-Generated Subjective Global Assessment [27]. Other assessments include body composition measured using bioelectrical impedance analysis (The Seca mBCA 515 Analyser) and a diet assessment (calories and protein) using a 24-hour diet recall.

Participants will receive a 200 mL bottle high protein oral nutritional supplement (Fresubin Protein Energy Drink, Fresenius Kabi) containing 20 g protein and 300 kcal per 200-mL bottle to be consumed daily within 60 minutes after exercise to support muscle synthesis [28-30]. Fresubin Protein Energy Drink is taken in addition to normal food and protein intake. Oral nutritional supplement tolerance will be assessed and recorded by the study dietitian. Dietary advice and written material will be provided with the aim of assisting the participant to achieve a protein intake (spread across meals) of 1.2-1.5 g per kg per body weight (or adjusted body weight in participants with obesity) as per the European Society for Clinical Nutrition and Metabolism guidelines [28]. Participants who have additional nutritional support needs (ie, malnutrition or nutrition-impact symptoms: early satiety, altered bowels, nausea, and loss of appetite), will have further individualized nutrition support and have education as a standard of care as determined by the dietitian and their clinical team. Nutrition support after surgery will be determined by the clinical team as per standard practice.

### Nursing Support

Participants will be contacted weekly by phone or in person, associated with gym visits, by a cancer nurse specialist to provide support promoting adherence to the exercise and nutritional interventions, reassurance, and to enable participants to discuss any concerns about their upcoming surgery. Participants who are smokers will be strongly encouraged to stop smoking. Sessions are based on semistructured questions developed in association with an experienced oncology clinical psychologist. See Multimedia Appendix 1 for further details. Participants could be referred to a clinical psychologist according to the standard of care if the cancer nurse specialist believed it was required.

### Program Satisfaction

Participants will complete an investigator-developed satisfaction survey at completion of the intervention prior to surgery (1-4 days before hospital admission or surgery) and 30 days after surgery (±7 days). A qualitative semistructured exit interview will be conducted 24-48 hours prior to hospital discharge and at 30 days after surgery, by an independent researcher face-to-face or over the phone in a subset of participants.

Surgical ward nurses will be requested to complete a survey before the prehabilitation intervention begins and after the completion of the study. Clinical specialists (surgeons and anesthetists) will be requested to complete a web-based evaluation survey after completion of the study.

### Study Outcomes

Data will be collected at 3 assessment times: baseline, before surgery (1-4 days before hospital admission or surgery), and 30 days (±7 days) after surgery assessment. Additional data will be collected during the participant’s hospital stay. Table 2 gives an overview of all assessments and measurements. The primary end point is related to implementation based on the RE-AIM evaluation framework (Table 1) and will be reported using the following dimensions:

- Reach: number (and proportion) of eligible patients having gastrointestinal cancer surgery who participated among eligible participants and patients who did not consent, and participant characteristics.
- Effectiveness: change in participant outcomes (functionality, nutrition, and well-being) before and after the intervention.
- Adoption: number (and proportion) of surgeons who referred and surgeon characteristics. Total number of referrals, referral sources, barriers to referrals, and clinician and surgeon perceptions.
- Implementation: delivery of the intervention as per the protocol and adaptations made. Overall completion rates, withdrawals and reasons, adherence to exercise sessions, nutrition intervention, and nurse support, and completion of program evaluations.
- Maintenance: status of the program 12 months after study completion and adaptions made.

The secondary end points collected are outlined in Table 2.

Other exploratory end points include the following:

- Participant satisfaction/experience: measured using an investigator-developed questionnaire and qualitative exit interviews.
- Compliance/adherence: measured through attendance logs (exercise and nursing support) and a participant self-reported diary (exercise and high protein oral nutritional supplement consumption).

**Table 2:** Assessments and outcomes.

Variables		Timeline
**Demographics/history**		
	Age Sex Smoking history Background, language spoken, with or without an interpreter Medical history Concomitant medications Cancer diagnosis Comorbidity index [31,32] Eastern Co-operative Oncology Group performance status [33]	Baseline, before surgery, and 30 days after surgery
**Functional assessment**		
	Handgrip strength 30-second chair stand [34] 2-minute step test [34] 3-m timed up and go [35] 6-minute walk test [19,20] Activities of daily living [36]	Baseline, before surgery, and 30 days after surgery
**Anthropometry**		
	Height (cm) (baseline only) Weight (kg) Body mass index (BMI) (kg/m^2^) Body composition: fat mass, fat-free mass, skeletal muscle mass, and visceral adipose tissue Waist circumference (cm)	Baseline, before surgery, and 30 days after surgery
**Nutritional assessment**		
	Nutritional status using PG-SGA^a^ [27] Total protein intake (diet and oral nutritional supplement): 24-hour diet recall Patient high protein supplement diary (during intervention)	Baseline, before surgery, and 30 days after surgery
**Patient-reported outcomes**		
	Exercise behavior—modified Godin’s Leisure Time Exercise Questionnaire [37] and Exercise Log/Diary QoL: EORTC QLQ-C30^b^ [33] FACIT-F^c^ subscale [38] Symptoms of anxiety and depression—HADS^d^ [39] Nutrition/Sarcopenia—PG-SGA. Short form [27] and sarcopenia screening using SARC-F^e^ [40] Distress—National Comprehensive Cancer Network Distress Thermometer (single item) [41]	Baseline, before surgery, and 30 days after surgery
**Adverse events**		
	Adverse events will be recorded and graded using National Cancer Institute Common Terminology Criteria for Adverse Events Version 5.0 (NCI CTCAE v5.0) [42]	Baseline and before surgery
**Hospital stay information**		
	Surgical complications: intra- and postoperative including Clavien Dindo Grading (I-V) [43] Length of hospital stay (days) Discharge information including medically cleared for discharge date, social services required on discharge, discharge destination, and so forth Length of physiotherapy intervention (days) Readmission within 30 days of surgery	30 days after surgery
**Compliance/adherence**		
	Attendance at exercise sessions Home-based exercise/protein diary (self-reported) Adherence to nurse calls Adherence to protein supplement drinks (self-reported)	Before surgery
**Program evaluation**		
	Investigator-developed program satisfaction survey Exit interview	Baseline and before surgery

^a^PG-SGA: Patient-Generated Subjective Global Assessment.

^b^EORTC QLQ-C30: European Organization for the Research and Treatment of Cancer Quality of Life Questionnaire.

^c^FACIT*-*F: Functional Assessment of Chronic Illness Therapy—Fatigue.

^d^HADS: Hospital Anxiety and Depression Scale.

^e^SARC-F: Strength, assistance with walking, rising from a chair, climbing stairs, and falls.

### COVID-19 Adaptations

Assessments and interventions were initially designed to be conducted face-to-face. In response to the COVID-19 pandemic, local lockdowns, reduction in nonurgent face-to-face health care, and cancellation of nonurgent elective surgery with decreased surgery activity, face-to-face recruitment, and intervention delivery were paused in March 2020. The study protocol was adapted to use digital technologies and telehealth methods, and recruitment was recommenced in August 2020. Textbox 2 highlights the key changes.

**Protocol telehealth adaptations to accommodate the COVID-19 pandemic.**

- Inclusion criteria
  Access to internet-based videoconferencing platforms including email address and internet (family members to assist if required)
- Consent
  Electronically completed consent forms
- Baseline, before surgery, and 30 days after surgery assessments
  Assessments conducted remotely over videoconferencing or zoom appointments with links emailed to participants
- Nutrition assessment
  Anthropometric variables self-measured: weight, height, and girths (waist, hip, and midarm; standardized tape measure provided)
  Body composition analysis omitted
- Exercise assessment
  Remote functional capacity assessment
    6-minute walk test and hand grip test omitted
    Continuation of 30-second chair stand, 2-minute step test, 3-m timed up and go (to measure cardiovascular fitness)
- Supervised exercise intervention
  Home-based individualized supervised exercise sessions conducted remotely via videoconferencing or zoom
  Principles of the original protocol were followed in terms of frequency, intensity, time, and types of exercise
- Nutrition intervention
  Education material and high protein oral nutritional supplements mailed to participants
- Patient-reported outcome questionnaires
  Completed by hard copy (mailed out) or web-based or electronically using Research Electronic Data Capture

### Statistical Analysis

#### Sample Size

The sample size is pragmatic, which is based on 2018/2019 surgical and oncology site numbers. We estimate that 50% (n=85) of eligible participants will agree to participate in the prehabilitation program. The study anticipates recruiting 100 participants over a 12-month period. Based on our pilot study adherence and the short-term nature of the intervention, we anticipate attrition will be less than 20% (n=20) of those who complete the baseline assessment, but higher in those who have already undergone neoadjuvant chemotherapy. This gives us sufficient statistical power at the 5% significance level to detect an increase of 20 m in the distance walked in the 6MWT from baseline to postintervention based on our pilot study.

#### Statistical Analyses

The primary outcome based on the RE-AIM evaluation framework will be reported descriptively, with a focus on referral, recruitment, and retention targets.

Secondary end points including baseline characteristics, functional, and nutritional status, will be reported using descriptive statistics, mean (SD) for normally distributed data, or median and IQR for nonnormally distributed data. Counts and percentages will also be used as appropriate. Reported changes in outcome variables from baseline to postintervention (presurgery) and 30 days after surgery will be measured using mixed models and will include all participants with baseline data including those with missing data at follow-up, with adjustment for predictors of dropouts to minimize selection bias. Postsurgery outcome variables will be evaluated using mixed models. Outcomes for UGI cancer and CRC participants will be evaluated separately and combined. Length of hospital stay and postsurgical complications will be compared to historical data from the hospital’s surgical 2019 database.

Responses to the investigator-developed questionnaire (participants and clinician) will be presented descriptively using means (SDs) or medians (IQR) and counts and percentages as appropriate. Free text responses will be summarized. The statistical package SPSS (version 24 or the newest release) will be used for quantitative data analysis.

Semistructured interviews regarding participant satisfaction will be transcribed verbatim and undergo qualitative content analysis. Initial transcript sample readings will be carried out. Themes and content will be analyzed descriptively. Coding, linking, and retrieving the qualitative data will be conducted using NVivo software (QSR International).

### Data Collection and Management

Participants will be given a unique study number. Deidentified information will be entered into a password-protected project specific Research Electronic Data Capture Database (Vanderbilt University) at Concord Cancer Centre, Concord Repatriation General Hospital. Identifiable information will be stored in a standalone instrument within the Research Electronic Data Capture database and access restricted to a limited number of the research team. In addition, deidentified raw body composition data will be stored on a copy of the manufacturer’s secure password-protected computer software (Seca) at the Concord Cancer Centre, Concord Repatriation General Hospital.

## Results

The study recruitment commenced in January 2020. Recruitment was paused in March 2020, due to the COVID-19 pandemic, and the protocol was adapted to include a remote or telehealth intervention. Recruitment restarted with the telehealth adaptations in August 2020. Due to ongoing COVID-19 disruptions (restrictions and cancellation of elective surgeries at the hospital), recruitment was extended until December 31, 2021. During the 16-month active recruitment period, a total of 198 (133 CRC and 65 UGI) patients were screened, of which 102 were ineligible. The most common reasons for ineligibility were less than a 14-day intervention time period between screening or referral and scheduled surgery date (n=64), not for surgical intervention (n=12), and not medically cleared to exercise (n=6). In total, 96 patients met the eligibility criteria, and 77 were recruited (64 patients with colorectal cancer and 13 patients with upper gastrointestinal cancer). The results from the study will be analyzed and published in a peer-reviewed journal.

## Discussion

### Principal Findings

Surgery remains the standard curative treatment for early-stage CRC and UGI cancers. Despite advancements in surgical practices and postoperative care pathways, morbidity and mortality rates remain high [6,7,10]. A reduction in postoperative complications is important. It is well established that the number and severity of postoperative complications are associated with preoperative health status, functional capacity, nutritional status, and muscle strength [44]. While traditional approaches have focused on lifestyle modifications in the postoperative period, recent evidence shows that the preoperative period may be the more appropriate time to intervene [45].

Positive lifestyle practices, including exercise and nutrition, are associated with many health benefits and have been shown to decrease the risk of several common cancers, including CRC and some UGI cancers, and possibly to decrease the risk of cancer recurrence [46,47]. However, only approximately 20% of people diagnosed with cancer are meeting the current exercise guidelines [48-50], and only 37% of patients with gastrointestinal cancer undergo nutrition screening and are offered preoperative nutrition support [51,52]. Recent reviews indicate that optimizing functional capacity with exercise, nutrition interventions, and psychological support strategies in a gastrointestinal surgical population resulted in reduced complications, length of hospital stay, and improved QOL [13-17]. However, few studies have evaluated the integration of prehabilitation practices into standard of care.

This pragmatic prehabilitation program will deliver integrated care, increasing patients’ self-management skills to support healthy living during and after a cancer diagnosis. It has the potential to change standard care with a simple, safe, relatively inexpensive coordinated intervention that confers multiple physical and psychological health benefits. This study will determine whether a multimodal (exercise, nutrition intervention, and nursing support) prehabilitation program can be integrated into routine standard care and whether it improves outcomes by reducing postoperative complication rates and length of stay at the hospital. Unique data will be collected on physical activity, nutritional status, dietary intake, and psychological well-being and their influence on surgical outcomes to inform the current evidence base in gastrointestinal surgical oncology preoperative care.

Prehabilitation adapted to include a telehealth model of care represents a strategy to combat pandemic-related patient and health system challenges. Telehealth initiatives provide an opportunity to minimize health inequalities [53] and improve patient outcomes and access to health care services [54] by offering an alternative delivery method that is not bound by location, especially for those living remotely, or those who have travel difficulties, caregiver or work commitments, or conflicting medical appointments. While telehealth interventions can vary from live consults, electronic monitoring, questionnaires, and support using multimedia platforms, the benefits of education sessions conducted by telehealth have shown to improve the patient’s knowledge and satisfaction during cancer treatment [55]. While the evidence in the preoperative setting is limited [56,57], preliminary findings indicate that home-based prehabilitation programs delivered by telehealth are positively received by patients and clinicians.

The hospital system is complex. Engagement from clinicians, administrative personnel, and participants is crucial to the successful implementation of a multimodal intervention. Importantly, the results of this study will identify priority areas regarding the value and feasibility of integrating prehabilitation into routine quality cancer care for patients undergoing major cancer surgery. It has the potential to improve the outcomes of patients undergoing resection for CRC and UGI cancer and deliver better health at a low cost to the Australian and wider communities.

### Conclusions

Prehabilitation represents an opportunity to intervene, treat, and prevent physiological and nutritional decline before major gastrointestinal cancer surgery by combining physical, nutritional, and psychological interventions in a multimodal approach. The study will provide new evidence on the integration of prehabilitation into standard care using adaptive models of health care delivery including telehealth, with the secondary aim of improving postoperative outcomes. The intervention could be generalizable to other types of cancer, emphasizing the importance of preoperative optimization to mitigate the surgical stress response and improve patient outcomes. The study outcomes have the potential to change standard care, with a relatively inexpensive intervention delivered either face-to-face or by telehealth especially for those living remotely or with travel or social difficulties, which can improve health-related quality of life and reduce health care costs.

## Appendix

Multimedia Appendix 1 Supplementary material.

## Abbreviations

6MWT: 6-minute walk test
CRC: colorectal cancer
QOL: quality of life
RE-AIM: Reach, Effectiveness, Adoption, Implementation, and Maintenance
UGI: upper gastrointestinal

## Data Availability

Data will be available from the corresponding author upon reasonable request.
