# Supplementary material for: Prehabilitation Before Gastrointestinal Cancer Surgery: Protocol for an Implementation Study
Source: JMIR Res Protoc. 2023 Mar 27;12:e41101. doi: 10.2196/41101 (PMC10131732; doi:10.2196/41101)
Supplement: Multimedia Appendix 1 [file resprot_v12i1e41101_app1.docx]

**Semi-structured questions for nurse support phone call.**

- How are you feeling?
- How are you coping with your participation in the prehab program?
- Do you have any concerns you would like to talk to me about?
- Have you taken your nutritional supplement within one hour of your exercise?
- Have you stopped smoking? (if relevant) Yes/No.
- If you are still smoking, how many cigarettes are you smoking per day?
- If you are still smoking, I would encourage you to stop smoking to improve your health and wellbeing for your future operation (Discuss nicotine patch, Quitline).
